# Protective Role of Catechin and Quercetin in Sodium Benzoate-Induced Lipid Peroxidation and the Antioxidant System in Human Erythrocytes *In Vitro*

**DOI:** 10.1155/2014/874824

**Published:** 2014-02-12

**Authors:** Gamze Yetuk, Dilek Pandir, Hatice Bas

**Affiliations:** Department of Biology, Faculty of Arts and Science, Bozok University, 66100 Divanli Yolu/Yozgat, Turkey

## Abstract

The aim of this study was to evaluate the protective effect of catechin and quercetin in sodium benzoate- (SB-) induced oxidative stress in human erythrocytes *in vitro*. For this, the effects of SB (6.25, 12.5, 25, 50, and 100 **μ**g/mL), catechin (10 **μ**M), and quercetin (10 **μ**M) on lipid peroxidation (LPO) and the activities of SOD, CAT, GPx, and GST were studied. Significantly higher LPO and lower activities of antioxidant enzymes were observed with the increasing concentrations of SB. Catechin or quercetin protected the erythrocytes against SB-induced toxicity only at low concentrations of SB. The presence of catechin or quercetin at 10 **μ**M have no effect on SB-induced toxicity at high concentrations of SB (50 and 100 **μ**g/mL). In conclusion, SB may cause oxidative stress as food additive in human erythrocytes *in vitro*. So, it appears that our findings provide evidence for the protection of erythrocytes from SB that could be considered for further studies.

## 1. Introduction

Food additives are chemical substances added to foods for various reasons to preserve flavor or enhance its taste and appearance. Various food additives are preservative in character. Preservatives are compounds that delay or prevent microbiological, enzymatic, or chemical changes of foods. They do not only act against visible spoilage by yeasts, molds, and bacteria but also prevent the formation of toxins, especially those produced by bacteria and molds [1]. They play an important role in the safety of food supply; many studies revealed the potential genotoxic and mutagenic effects of the additives [2–5]. Consumption of food additives has caused various diseases such as eczema, urticaria, diarrhea, nausea, migraine, and vomiting [6].

Sodium benzoate is widely used as food preservatives [7] on salad dressings, carbonated drinks, jams, and fruit juices [5]. The acceptable daily intake (ADI) levels recommended by the Joint FAO/WHO Expert Committee on Food Additives (JECFA) for sodium benzoate and potassium benzoate are 0–5 mg/kg body weight [5, 8]. In general the sodium and potassium salts are preferred over the acid form because they are more soluble in water [9].

Flavonoids are polyphenolic compounds found in significant quantities in the human diet [10, 11]. The flavonoids are potent antioxidants *in vitro* [12, 13]. Quercetin, a flavonoid found in fruits and vegetables [14], exerts beneficial effects [15] that contribute to human health. The antioxidant effect by scavenging free radicals and chelating iron ions of quercetin has been extensively studied [12, 16] because free radicals are involved in pathogenesis of many diseases [17]. Catechins are flavonoids that have particularly attracted attention because of their relatively high antioxidant capacity. Green and black tea contain considerable amounts of catechins and several studies have attributed the *in vitro* antioxidant properties of tea extracts to the presence of these flavonoids [18, 19].

According to the literature survey, no study has been conducted on enzyme activities and LPO of sodium benzoate (SB) + catechin and SB + quercetin in human erythrocytes using spectrophotometric analysis *in vitro.* For these reasons, the aim of this study was to investigate the effect of SB in human erythrocytes as* in vitro*; then malondialdehyde (MDA) levels, SOD, CAT, GPx, and GST activities were assessed and undertaken to determine whether the free radical scavengers catechin or quercetin could inhibit the oxidative destruction of unsaturated fatty acids and alter enzymatic activities.

## 2. Materials and Methods

### 2.1. Chemicals

SB (99% purity) was obtained from Merck (Germany). Quercetin and catechin were supplied by Sigma-Aldrich (Germany), and dimethyl sulfoxide (DMSO) was supplied by Merck (Germany). SB was dissolved in distilled water [5] and quercetin and catechin were dissolved in 0.5% DMSO [20]. All other chemicals used were analytical grade and were of obtained from Sigma-Aldrich (Germany).

### 2.2. Treatment of Erythrocytes

Blood was collected from normal healthy volunteers into tubes containing heparin approximately 20 mL of fresh blood. Erythrocytes were separated from blood plasma by centrifuge and were divided into nontreated control and experimental groups. The control group was incubated in 0.9% NaCl at pH 7.4. Experimental groups were divided into treatment groups: SB (*n* = 6), catechin (*n* = 6), quercetin (*n* = 6), SB + catechin (*n* = 6), and SB + quercetin (*n* = 6) groups. The control group was incubated for 1 h at 37°C in 0.9% NaCl [21–23]. Erythrocytes in the experimental group were treated with 6.25, 12.5, 25, 50, and 100 *μ*g/mL of SB in the presence or absence of catechin (10 *μ*M) and quercetin (10 *μ*M) for 1 hr at 37°C. After incubation, the cell mixtures were stored at −20°C for 24 h. The mixtures were thawed; the erythrocytes were destroyed by osmotic pressure and then subjected to centrifugation. Supernatants were isolated and MDA levels and the activities of SOD, CAT, GPx and GST, were measured by spectrophotometer (Shimadzu UV-1800, Japan). The concentration of hemoglobin was determined using the method of Drabkin [24].

### 2.3. Lipid Peroxidation in Erythrocytes

LPO was measured in erythrocytes as MDA whose content was assayed by using the thiobarbituric acid test as described by Ohkawa et al. [25]. The absorbance at 532 nm was measured by a spectrophotometer. Specific activity is presented as nmol/mg hemoglobin.

### 2.4. Antioxidant Enzymes in Erythrocytes

SOD, CAT, GPx, and GST enzyme activities were determined in erythrocytes lysate prepared according to the method of McCord and Fridovich (1969) [26]. SOD was assayed according to the technique of S. Marklund and G. Marklund (1974) [27]. One unit of SOD activity was calculated as the amount of protein causing 50% inhibition of pyrogallol autooxidation. Activity was monitored at 440 nm. Data were expressed as USOD/mg hemoglobin. CAT was estimated according to the method of Aebi (1984) [28] and rate of decomposition of hydrogen peroxide by CAT enzyme at 240 nm was evaluated. CAT activity was read at 240 nm and expressed as UCAT/mg hemoglobin. GPx activity was measured by the method described by Paglia and Valentine (1967) [29]. It catalyses the oxidation of glutathione (GSH) by cumene hydroperoxide. GPx activity was read at 340 nm and expressed as UGPx/mg hemoglobin. GST activity was measured according to the method of Habig et al. (1974) [30]. The principle of the assay is based on the determination of the formation of GSH and the 1-chloro-2,4-dinitrobenzene conjugate. Increases in absorbance were recorded at 340 nm. The specific activity of GST is expressed as UGST/mg hemoglobin.

### 2.5. Statistical Analysis

The data are expressed as the means ± standard deviation (SD). Differences among experimental groups were analyzed by software program SPSS 11.0 for Windows. Comparison between means was carried out using one-way analysis of variance (ANOVA), followed by Tukey's procedure for multiple comparisons. *P* < 0.05 was taken as statistically significant.

## 3. Results

**Figure 1:**
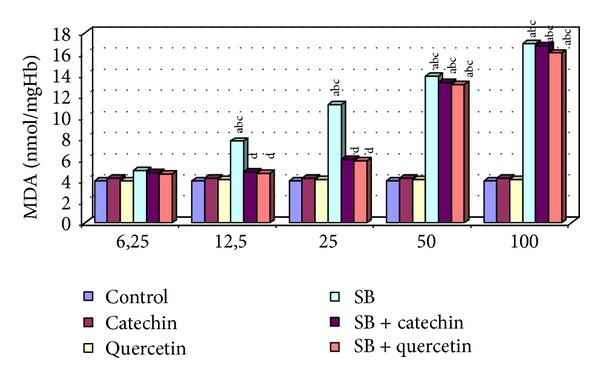
MDA level in erythrocytes of control and experimental groups. Different letters above bars indicate significant differences between exposure concentrations; bars with the same letter are not significantly different. Values are mean ± SD in each group. Significance at *P* < 0.05.

**Figure 2:**
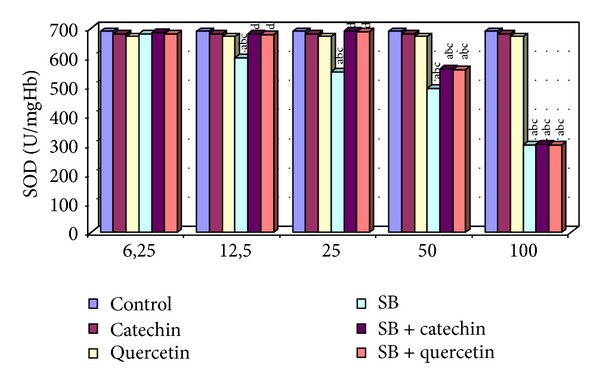
SOD activity in erytrocytes of control and experimental groups. Different letters above the bars indicate significant differences between exposure concentrations; bars with the same letter are not significantly different. Values are mean ± SD in each group. Significance at *P* < 0.05.

**Figure 3:**
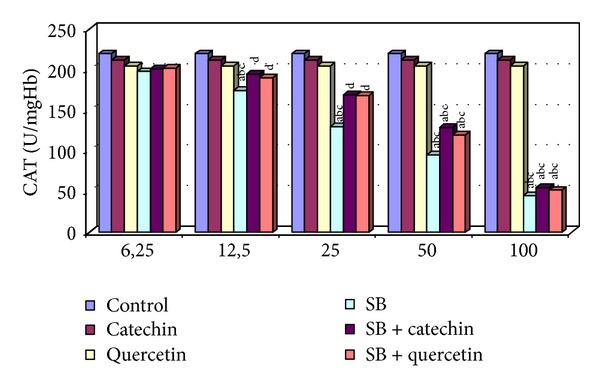
CAT activity in erytrocytes of control and experimental groups. Different letters above the bars indicate significant differences between exposure concentrations; bars with the same letter are not significantly different. Values are mean ± SD in each group. Significance at *P* < 0.05.

**Figure 4:**
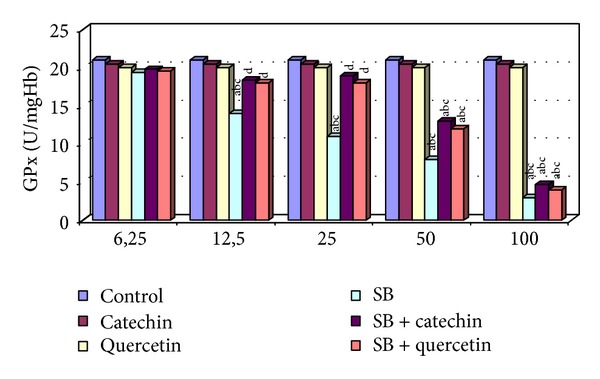
GPx activity in erytrocytes of control and experimental groups. Different letters above the bars indicate significant differences between exposure concentrations; bars with the same letter are not significantly different. Values are mean ± SD in each group. Significance at *P* < 0.05.

**Figure 5:**
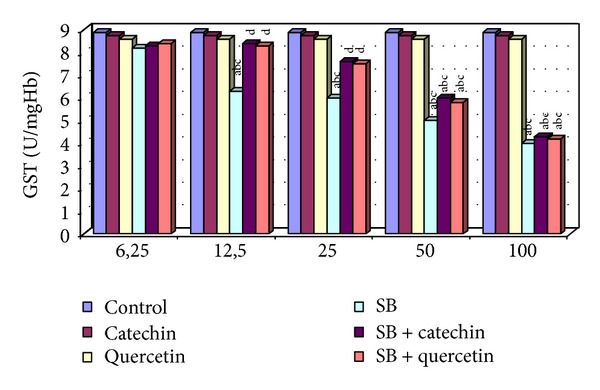
GST activity in erytrocytes of control and experimental groups. Different letters above the bars indicate significant differences between exposure concentrations; bars with the same letter are not significantly different. Values are mean ± SD in each group. Significance at *P* < 0.05.

There were no statistically significant changes in MDA levels and in SOD, CAT, GPx, and GST activities between the catechin- and quercetin-treated groups compared with the control group (*P* > 0.05, Figures 1–5).

### 3.1. Measurement of Level of Malondialdehyde (MDA)

SB treatment (12.5, 25, 50, and 100 *μ*g/mL) increased significantly the level of MDA compared with control, catechin, and quercetin-treated groups (*P* < 0.05). In contrast, any change of MDA level was not detected at only the lowest dose of SB (6.25 *μ*g/mL). The MDA levels were decreased statistically significantly in the SB + catechin treated group and SB + quercetin-treated group compared to SB-treated group (*P* < 0.05, Figure 1). Decreasing the MDA level of 50 *μ*g/mL of SB incubated with catechin or quercetin group was measured not to be statistically significant (*P* > 0.05). The protective effect of catechin or quercetin was not detected in the 100 *μ*g/mL SB-treated group in human erythrocytes *in vitro.*

### 3.2. Measurement of Superoxide Dismutase (SOD) Activity

Decreases in SOD activity were found in human erythrocytes in used high four concentrations. No differences in the activity of SOD were observed in treated erythrocytes at the lowest dose of SB-(6.25 *μ*g/mL). The SOD activity was increased statistically significantly in the SB + catechin treated group and SB + quercetin treated group compared to SB (12.5 and 25 *μ*g/mL) treated group (*P* < 0.05, Figure 2). Increasing the SOD activity of 50 *μ*g/mL of SB incubated with catechin or quercetin group was measured not to be statistically significant (*P* > 0.05). The protective effect of catechin or quercetin was not detected in the 100 *μ*g/mL SB-treated group in human erythrocytes *in vitro.*

### 3.3. Measurement of Catalase (CAT) Activity

Any alternation of CAT activity was not detected in the human erythrocytes in 6.25 *μ*g/mL, decreased significantly in 12.5, 25, 50, and 100 *μ*g/mL compared with control groups (*P* < 0.05). Catechin or quercetin supply combined with SB partially reversed this change only at 12.5 and 25 *μ*g/mL doses of SB. CAT activity in 50 *μ*g/mL SB + catechin and 50 *μ*g/mL SB + quercetin groups was significantly higher than SB-exposed erythrocytes but it was not detected to be statistically significant compared to control, catechin, and quercetin groups (*P* > 0.05, Figure 3). The protective effect of catechin or quercetin was not detected in 100 *μ*g/mL SB-treated group in human erythrocytes *in vitro.*

### 3.4. Measurement of Glutathione Peroxidase (GPx) Activity

As shown in Figure 4, the GPx activity was significantly decreased in high doses of SB-exposed erythrocytes, whereas 6.25 *μ*g/mL of SB was no change this enzyme activity as compared to control, catechin and quercetin groups. Catechin or quercetin supply in the SB-treated group significantly increased GPx activity in erythrocytes compared to control groups only at 12.5 and 25 *μ*g/mL SB (*P* < 0.05, Figure 4). Catechin or quercetin has no protective effect on SB-induced changes in GPx activity in erythrocytes at 50 and 100 *μ*g/mL doses of SB.

### 3.5. Measurement of Glutathione-S-Transferase (GST) Activity

The activity of GST was significantly decreased in the SB-treated (12.5, 25, 50, and 100 *μ*g/mL) groups, and, in contrast, no statistically significant changes were observed in the 6.25 *μ*g/mL of SB compared with the control, catechin, and quercetin groups. However, the GST activity was significantly increased in erythrocytes in the SB + catechin and SB + quercetin groups compared with the SB-only group (*P* < 0.05, Figure 5). There were no statistical differences among 50 *μ*g/mL SB + catechin or quercetin-treated erythrocytes compared to only SB-treated cells. Catechin or quercetin has no protective effect on SB-induced changes in GST activity in erythrocytes at 100 *μ*g/mL dose of SB.

## 4. Discussion

Different increasing doses of SB were tested by using chromosome aberrations, sister-chromatid exchanges, and micronucleus in cultured human lymphocytes and comet assay in isolated human lymphocytes. The results show that SB is genotoxic to the human peripheral blood lymphocytes *in vitro* at the highest concentrations [5]. So, potential genotoxic effects of food preservatives SB was used in the present study same concentrations that by the previous works reported in human blood *in vitro*.

Piper (1999) [31] says that SB can create free radicals and damage cells. SB intoxication showed increased erythrocyte LPO associated with lowered antioxidant enzymes; as the activities of SOD, CAT, GPx, and GST decreased, the MDA level increased. The mechanism of the effect of SB on enzyme activities and MDA level of erythrocytes may be as well as many chemical substances [32]. The decrease in SOD, CAT, GPx, and GST activities in erythrocytes may be due to the inactivation of these enzymes, as superoxide anions have been shown to reduce the activity of these enzymes [33]. The depletion of erythrocyte GSH along with the decrease in SOD and CAT activities may affect the ability of erythrocytes to scavenge superoxide anions and hydroxyl radicals. The decrease of GSH content in erythrocytes by SB intoxication may be due to depletion of GSH, SB-SH binding, or an effect on glutathione reductase activity [32].

The antioxidants protect the erythrocyte membrane from oxidative damage [34, 35] and also prevent oxidative damage as a result of their ability to scavenge reactive oxygen species such as hydroxyl radical and superoxide anion [36]. Studies in animals and humans suggest that flavonoids may reduce the risk of cardiovascular diseases [11, 37, 38], cerebrovascular diseases [39], and cancers [40]. Catechins are flavonoids that are found in green tea, black tea, and other plant foods and they are reported to have various physiological effects in terms of their antioxidative ability [20, 41]. The protective effects of myricetin, quercetin, (+)-catechin, and (−)-epicatechin were investigated against N-nitrosodibutylamine and N-nitrosopiperidine-induced DNA damage in HepG2. (+)-catechin at the lowest concentration (10 *μ*M) showed the maximum reduction of DNA strand breaks [42]. Lotito and Fraga (2000) [43] have shown that (+)-catechin was an effective antioxidant in human blood plasma, delaying the consumption of endogenous lipid soluble antioxidants and inhibiting lipid oxidation. Quercetin, a potent antioxidant, scavenges free radicals directly, inhibits LPO, and alters antioxidant defence pathway *in vivo* and *in vitro* [44]. Quercetin, a naturally occurring flavonoid, is able to protect RPE cells from oxidative damage and cellular senescence *in vitro* dose-dependent manner [45]. (+)-catechin and quercetin at the lowest concentration (10 *μ*M) were used against SB-induced oxidative stress in human erythrocytes *in vitro* in this study. Our results indicated that the presence of (+)-catechin and quercetin at 10 *μ*M concentration could be able to ameliorate SB-induced oxidative stress by decreasing LPO and altering antioxidant defense system in erythrocytes.

In conclusion, the effects of SB, catechin, and quercetin on LPO and the antioxidant enzyme systems were evaluated in human erythrocytes *in vitro* in this study. High concentrations of SB may induce oxidative stress by enhancing LPO and by altering the antioxidant enzyme systems in erythrocytes. We observed that the antioxidant enzyme activities and MDA levels were ameliorated in the catechin or quercetin + SB treated groups. These protective effects may be due to antioxidant effects of catechin and quercetin. So, our findings suggest that using of SB as food preservatives caution be taken and should be preferred lowest concentrations of SB.
